# Platelet Factor 4 Regulation of Monocyte KLF4 in Experimental Cerebral Malaria

**DOI:** 10.1371/journal.pone.0010413

**Published:** 2010-05-03

**Authors:** Kalyan Srivastava, David J. Field, Angela Aggrey, Munekazu Yamakuchi, Craig N. Morrell

**Affiliations:** Aab Cardiovascular Research Institute, University of Rochester School of Medicine and Dentistry, Rochester, New York, United States of America; Leiden University Medical Center, Netherlands

## Abstract

Cerebral malaria continues to be a difficult to treat complication of *Plasmodium falciparum* infection in children. We have shown that platelets can have major deleterious immune functions in experimental cerebral malaria (ECM). One of the platelet derived mediators we have identified as particularly important is platelet factor 4/CXCL4. Our prior work demonstrated that PF4^−/−^ mice are protected from ECM, have reduced plasma cytokines, and have reduced T-cell trafficking to the brain. We now show that PF4 drives monocyte cytokine production in a Kruppel like factor 4 (KLF4) dependent manner. Monocyte depleted *Plasmodium berghei* infected mice have improved survival, and KLF4 is greatly increased in control, but not monocyte depleted mice. PF4^−/−^ mice have less cerebral monocyte trafficking and no change in KLF4 expression. These data indicate that PF4 induction of monocyte KLF4 expression may be an important step in the pathogenesis of ECM.

## Introduction

Cerebral malaria is a major complication of *Plasmodium falciparum* infection in children. In a recent study 33% of children admitted to a hospital in Kenya were reported to have had malaria, and of these, 47% had neurologic symptoms [1]. In 2002 alone there were an estimated 515 million clinical episodes of acute *P. falciparum* infection worldwide, mainly affecting children less than 5 years of age [1]. Cerebral Malaria (CM) is the result of a combination of vascular and immune system dysfunction. Brain tissue from patients that die of CM reveals multifocal capillary obstruction with parasitized red blood cells (RBC), platelets and leukocytes [2]. Several hypotheses have attempted to explain the noted pathology, but most now include cell adhesion to the endothelium or direct infected RBC (iRBC) interactions with platelets as promoting pro-thrombotic immune responses, resulting in further vascular inflammation, immune stimulation and obstruction of cerebral capillaries [2].

In addition to their vital role in hemostasis, platelets are also active in inflammation [3]. Platelet granules contain many inflammatory and adhesion molecules that are either released or expressed upon activation and platelets can initiate interactions with quiescent endothelial and immune cells [4]. Platelets are known to contribute to the progression of diverse vascular and inflammatory diseases including the pathogenesis of CM [5]–[7]. Platelet and RBC aggregates are found in cerebral blood vessels of individuals with fatal CM [7], [8]. Cytokines, such as TNFα and IL-6, are also greatly increased in the disease course and cytokine dysregulation has a major role in the progression of CM. TNFα can increase platelet binding to the brain microvasculature in ECM [9], further demonstrating this important interplay between platelets and immune responses in cerebral malaria.

We have demonstrated using the *Plasmodium berghei* ANKA mouse model of experimental cerebral malaria (ECM) that platelets are activated by direct CD36 dependent interactions with *Plasmodium* iRBCs leading to increased circulating levels of platelet factor 4 (PF4/CXCL4) [10]. PF4^−/−^ mice have greatly improved survival and reduced plasma chemokine and cytokine concentrations [10]. A prominent function of chemokines is to promote the chemotaxis and activation of leukocytes. PF4 was the first described CXC class chemokine and is abundant in platelet granules (about 25% of total alpha granule protein content) [11], [12]. PF4 can exert effects on monocytes, neutrophils, NK cells, and T-cells through poorly defined mechanisms [13], [14] and we have described a reduction in T-cell trafficking in *Plasmodium berghei* (*P. berghei*) infected PF4^−/−^ mice compared to WT mice [10]. Although chemokine signaling is an important mediator of CM [15], [16], there has been little examination of the role for platelet derived chemokines. Basal PF4 concentration in human plasma is 2–20 ng/mL, but in acute thrombosis has been reported elevated over 1000 times to 5–10 µg/mL [11], [17]. During acute *P. falciparum* infection in humans plasma concentrations of PF4 are also elevated [18] and we have shown that plasma PF4 is greatly increased in ECM (over 1 µg/mL), demonstrating a potentially significant role for platelets and PF4 in mediating malaria associated immune dysregulation.

The role of T-cells in ECM is well explored, but monocytes/macrophages have received much less attention despite a potentially important role in driving innate and initiating acquired immune responses. Platelet-monocyte interactions are well established in other vascular inflammatory diseases such as atherosclerosis [3], [19]–[21], but have not been studied in the context of ECM. PF4 can induce monocyte cytokine production [22]. Kruppel like factor 4 (KLF4) is a transcription factor necessary for monocyte differentiation and acquisition of an inflammatory phenotype [23]–[25]. We now demonstrate both *in vitro* and in ECM that PF4 increases monocyte cytokine stimulation, which is dependent at least in part, on PF4 increasing the expression of the transcription factor KLF4.

## Results

### PF4 induces monocyte cytokine production

**Figure 1:**
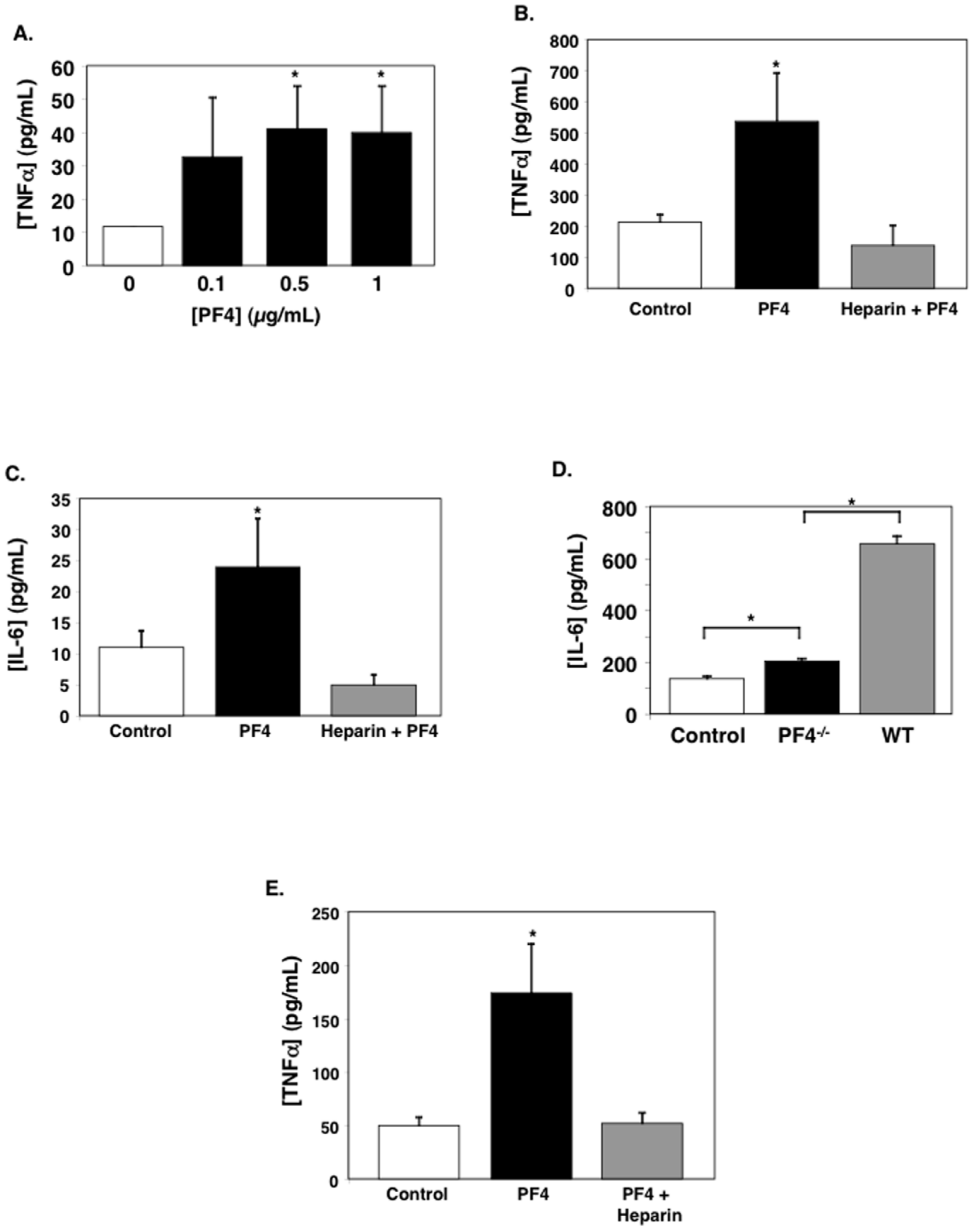
PF4 Stimulates Monocyte Activation. A. Dose Response. Monocytes were incubated with PF4 for 48 hrs and TNFα measured by ELISA (n = 4 ± S.D. *P<0.03 vs 0). B. Monocytes were treated with control PBS, 1 µg/mL PF4, or 50 U/mL heparin prior to PF4 as control. TNF-α was measured 48 hrs later (n = 4 ± S.D. *P<0.01 vs Control). C. IL-6. IL-6 was measured 48 hrs after PF4 by ELISA (n = 4 ± S.D. *P<0.01 vs control). D. Platelet PF4 increases monocyte IL-6 production Mouse monocytes were incubated with resting WT platelet supernatant and activated WT or PF4^−/−^ platelet releasate. 24 hrs later IL-6 was measured (n = 4 ± S.D. *P<0.01). E. Human monocyte cell line. THP-1 cells were incubated with 1 µg/mL of PF4 or PF4 in the presence of heparin and TNFα production measured (n = 3 ± S.D. *P<0.01 vs Control).

PF4 has been described as a mediator of monocyte differentiation and cytokine production [26]. A major outcome of monocyte activation is elaboration of inflammatory mediators such as TNFα and IL-6, each of which has been shown to be necessary or associated with cerebral malaria [8], [27]–[30]. Our prior studies have shown that in ECM PF4^−/−^ mice have greatly reduced plasma TNFα compared to WT mice [10]. To demonstrate that PF4 stimulates monocyte TNFα production we isolated primary marrow derived monocytes from mice, incubated these cells with physiologic concentrations of recombinant PF4 for 48 hrs (we have shown that PF4 levels are greater than 1 µg/mL in ECM [10]) and TNFα production was measured in the supernatant using an ELISA. PF4 drives increased monocyte TNFα production (Figure 1A). To demonstrate that this is a direct PF4 effect, and not the result of recombinant protein contamination, we incubated primary mouse monocytes with control buffer, 1 µg/mL of recombinant PF4, or PF4 in the presence of 50 U/mL of heparin to neutralize the PF4. PF4 again greatly increased TNFα production, but this was inhibited in the presence of heparin (Figure 1B). Similarly, PF4 also increased mouse monocyte IL-6 production (Figure 1C).

Platelets can stimulate monocytes by both contact dependent and independent mechanisms [4], [19], [20]. To demonstrate the importance of PF4 in platelet releasate mediated monocyte activation, we isolated WT and PF4^−/−^ mouse platelets and incubated each platelet type with 0.5 U/mL of thrombin for 10 mins to fully activate, before neutralizing with an equal concentration of hirudin. Platelet releasate was then isolated and incubated with monocytes for 24 hrs at a physiologic ratio of releasate source platelets to monocytes (releasate from 2×10^6^ platelets added to 1×10^5^ monocytes). IL-6 was measured using an ELISA. PF4^−/−^ platelet releasate significantly increased monocyte IL-6 production (Figure 1D grey bar). However, WT platelet releasate induced a much greater increase in monocyte IL-6 production (Figure 1D, black bar). These data directly indicate that PF4 has a major role in platelet induced monocyte cytokine production either alone, or in combination with other platelet immune mediators that it may complex with. To confirm that these data were not an artifact of mouse primary monocyte isolation, the human monocyte cell line, THP-1 cells, were incubated with 1 µg/mL of human platelet isolated PF4. PF4 also induced an increase in THP-1 TNFα production (Figure 1E). These data demonstrate that concentrations of PF4 relevant to ECM induce monocyte cytokine production, including IL-6 and TNFα.

### PF4 increases monocyte KLF4 expression and activity

**Figure 2:**
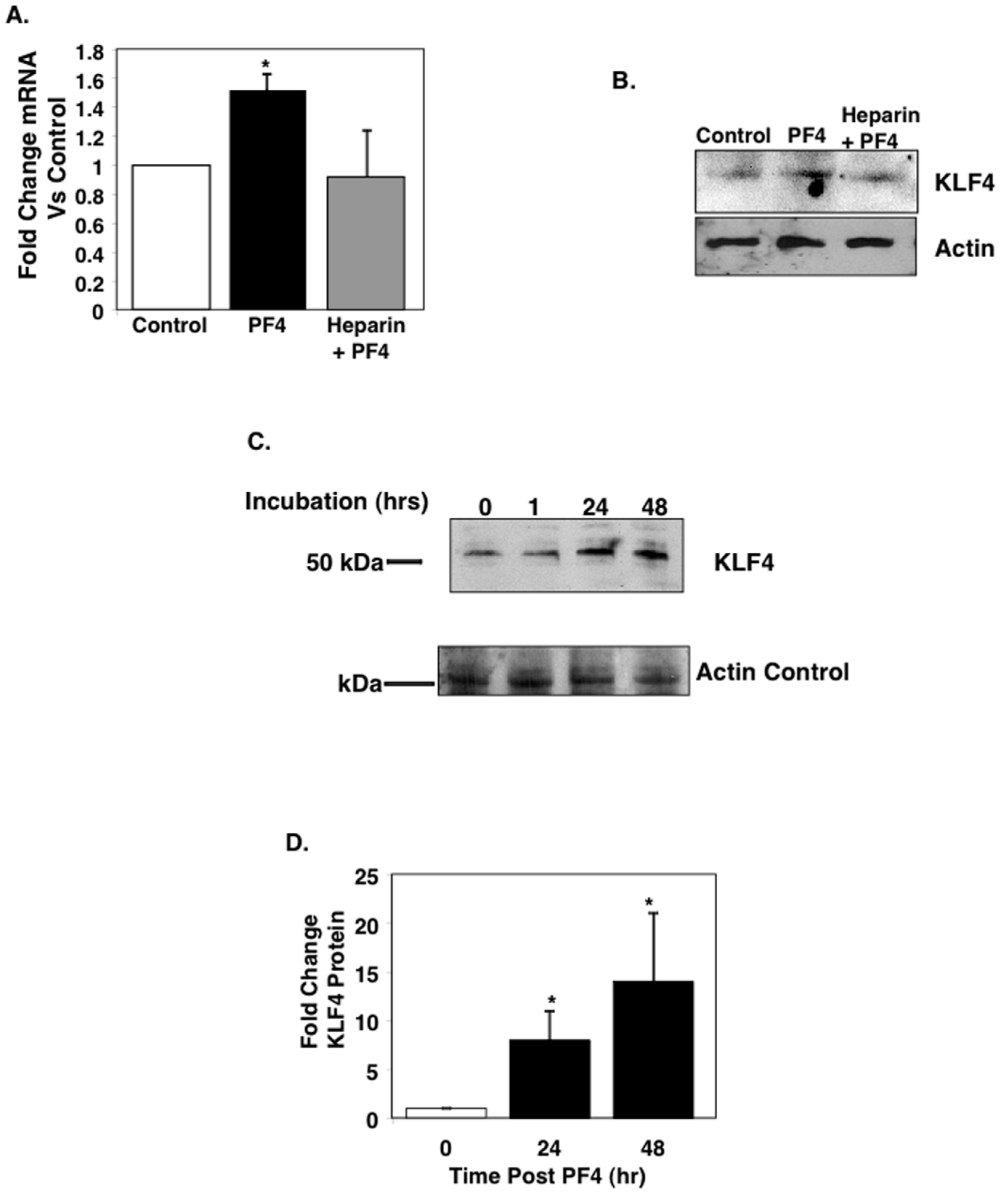
PF4 Increases Monocyte KLF4 Expression. A. Mouse monocytes were incubated with 1 µg/mL of PF4 and KLF4 mRNA was quantified by qRT-PCR (n = 4; ± S.D. *P<0.05 vs Control). B. KLF4 immunoblot (n = 4 pooled samples). C. KLF4 Time Course. THP-1 cells were incubated with 1 µg/mL of PF4 and at each time point KLF4 expression was determined by immunoblot (representative image). D. Western blot quantification of KLF4 (n = 3; ± S.E.M *P<0.05 vs Time 0).

KLF4 is a transcription factor vital to monocyte development, differentiation, and pro-inflammatory phenotype [23], [25]. KLF4^−/−^ mice are embryonic lethal and mice reconstituted with KLF4^−/−^ bone marrow have greatly reduced monocyte numbers [23]. To begin to determine if PF4 mediated effects on monocytes are KLF4 dependent, we isolated mouse monocytes and incubated these cells with control PBS, PF4, or PF4 after heparin pre-treatment. KLF4 mRNA expression was then determined 24 hours later by quantitative Real-Time PCR (qRT-PCR). PF4 significantly increased KLF4 mRNA (Figure 2A). This was confirmed by Western blot on monocytes treated the same way and demonstrating that PF4 also increased KLF4 protein expression (Figure 2B). (These experiments were also performed after de-glycosylating monocytes with chondroitnase ABC, but the treatment itself induced KLF4 expression, perhaps the result of the cleavage establishing downstream signaling and KLF4 expression). We also incubated THP-1 cells with 1 µg/mL of PF4 and at 1, 24, and 48 hrs determined KLF4 expression by Western blot. KLF4 expression was increased 24 and 48 hrs of incubation with PF4 (Figure 2C and quantification Figure 2D; no change at 72 hrs compared to control not shown). These data demonstrate that PF4 drives an increase in KLF4 expression.

**Figure 3:**
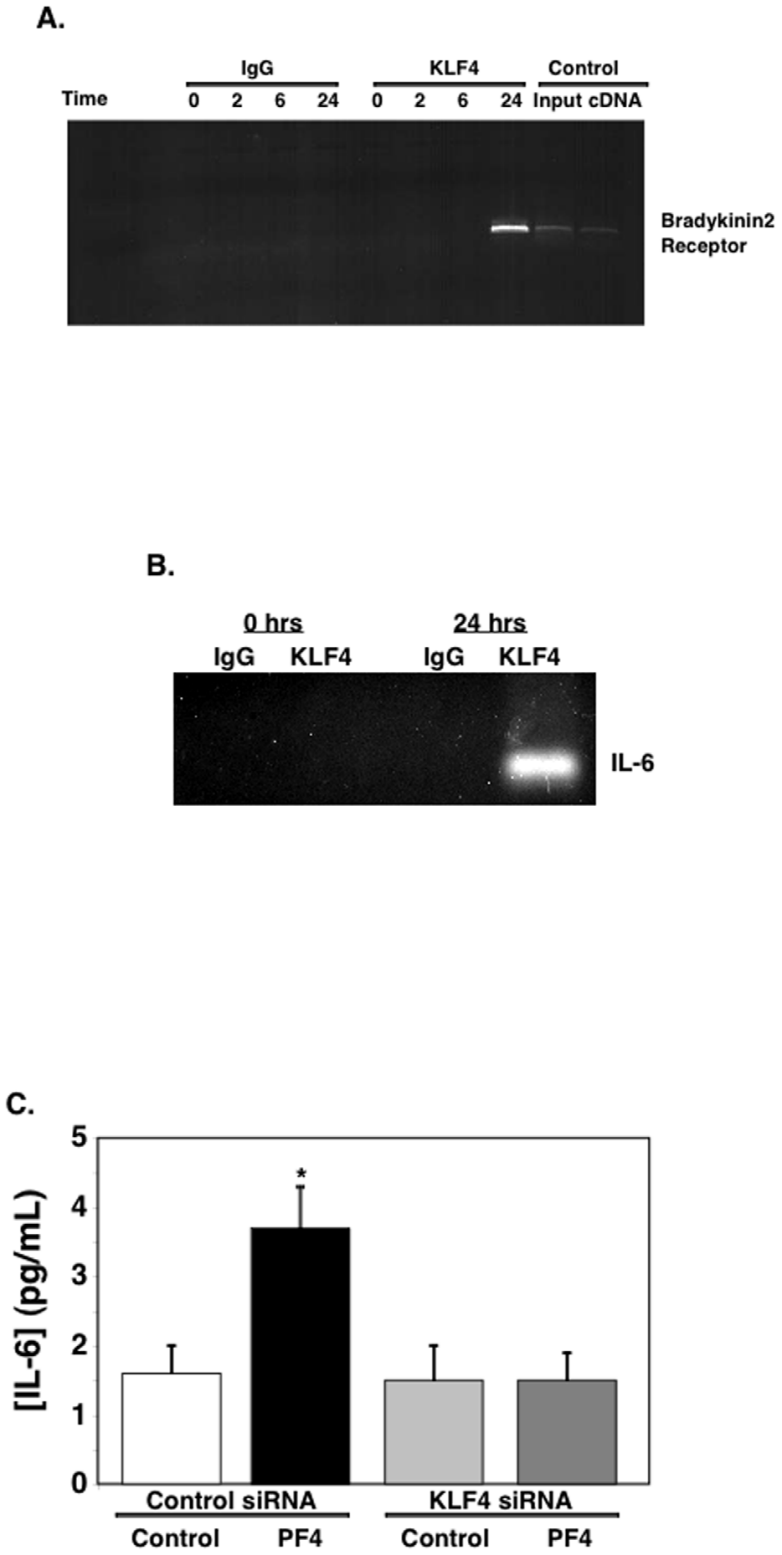
PF4 Stimulates KLF4 Activity. A. PF4 induces KLF4 DNA binding. THP-1 cells were incubated with 1 µg/mL of PF4 and at each time point nuclear extracts isolated for ChIP. DNA was prepared and PCR amplified with primers for the known positive control bradykinin 2 receptor promoter. Control DNA and control total spleen cDNA were used as positive controls (far right). B. IL-6 Promoter Binding. KLF4 ChIP DNA was prepared and PCR amplified with primers for the IL-6 promoter. C. KLF4 mediates PF4 induced monocyte stimulation. THP-1 cells were treated with control siRNA or KLF4 siRNA and incubated or not with 1 µg/mL PF4. IL-6 was measured by ELISA (n = 4 ± S.D. *P<0.01 vs control).

Because KLF4 is a transcription factor, PF4 induced increase in KLF4 expression is expected to increase KLF4 DNA binding. To demonstrate this, THP-1 cells were incubated with 1 µg/mL of PF4 and 0, 2, 6 and 24 hrs later nuclear extracts were isolated for chromatin immunoprecipitation (ChIP). DNA was prepared and PCR amplified using primers for the known KLF4 binding promoter sequence of the bradykinin 2 receptor promoter. Control kit DNA and control total spleen cDNA were used as positive controls (far right). At 24 hrs post PF4 addition KLF4 immunoprecipitates with genomic DNA, but not at the early time points we examined (Figure 3A). Because PF4 drives an increase in IL-6 and TNFα production, we also amplified immunoprecipitated DNA with primers for the promoter regions for both IL-6 and TNFα. IL-6 promoter DNA immunoprecipitated with KLF4 (Figure 3B), but not TNFα (not shown). This indicates that PF4 may directly increase IL-6 through KLF4, but PF4 induced increase in TNFα is indirect/downstream or through other transcription factor pathways.

Having established that PF4 induced monocyte cytokine signaling and KLF4 expression, we wanted to demonstrate the two events were functionally linked. To do so, we treated THP-1 cells with control siRNA or siRNA specific for KLF4 to knockdown KLF4 expression (Supplementary Figure S1). We then treated the cells with buffer or 1 µg/mL of PF4 and measured IL-6 production by ELISA 24 hrs later. PF4 increased IL-6 production in control siRNA treated cells, but had no effect on IL-6 production from KLF4 specific siRNA treated cells (Figure 3C). These data demonstrate that PF4 induction of monocyte activation is KLF4 dependent.

### PF4 increases monocyte KLF4 in ECM

**Figure 4:**
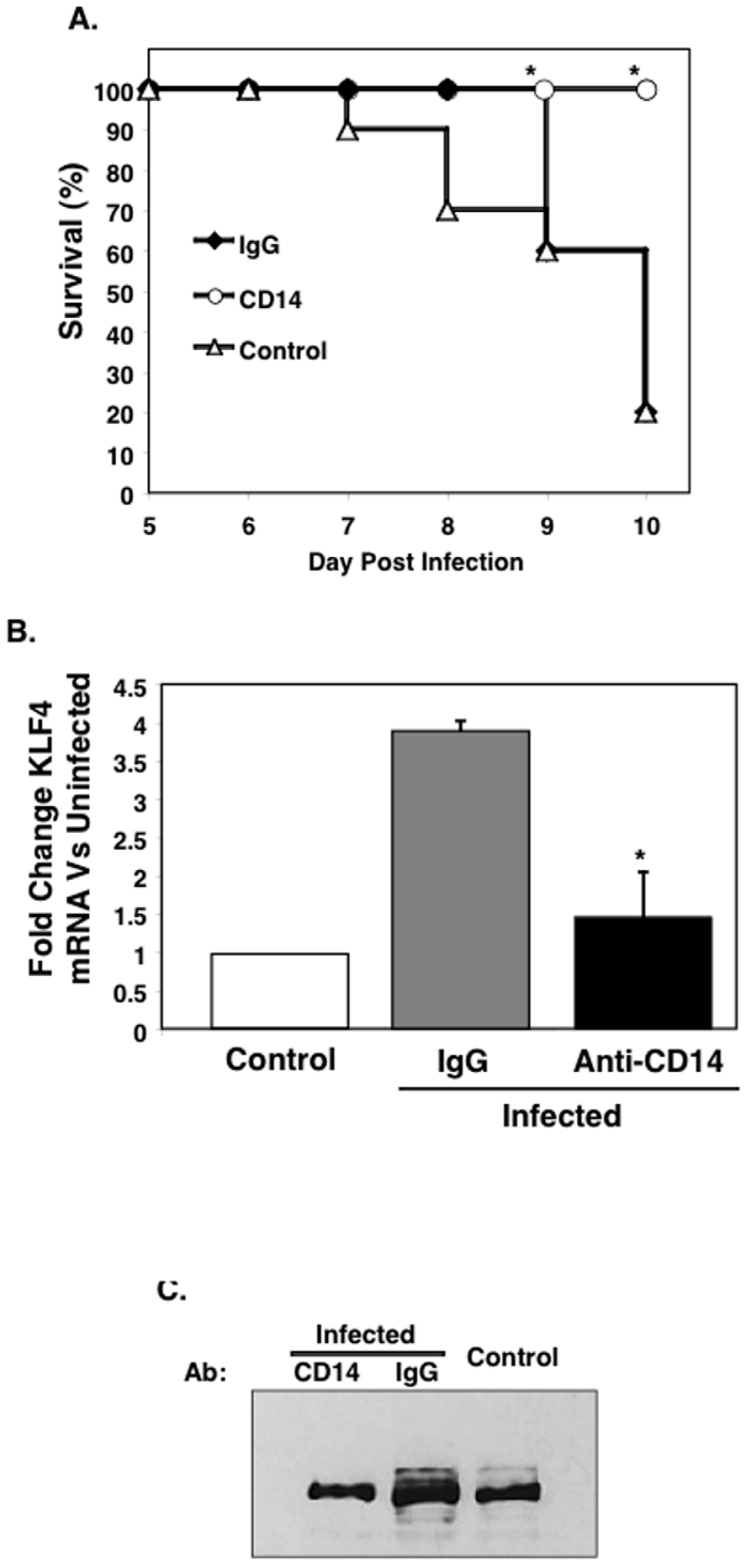
Monocytes are Important in the Development of ECM. A. Survival Curve. Mice were monocyte depleted with anti-CD14 antibody and infected with *P. berghei* (n = 5 *P<0.05 vs IgG). B. Monocytes are a significant source of KLF4 in ECM. mRNA. qRT-PCR for KLF4 was performed on spleens of uninfected and infected IgG and anti-CD14 treated mice (n = 5, S.D. *P<0.01 vs IgG) C. Protein. Spleen lysates were pooled and immunoprecipitated with anti-KLF4 antibody prior to KLF4 immunoblot (n = 5 mice).

We next sought to clearly demonstrate that monocytes have a key role in the pathogenesis of ECM. Mice infected with P. berghei ANKA were injected intraperitoneal with either control PBS, IgG, or anti-CD14 antibody (days 1 and 5 post infection) to clear monoctyes from the circulation without affecting other blood cells (complete blood counts in Supplementary Figure S2). Mice were then monitored for survival. Monocyte depleted mice had significantly improved survival (Figure 4A) confirming that monocytes are important in the pathogenesis of ECM.

Our in vitro data demonstrated that KLF4 is important in PF4 mediated monocyte activation. To demonstrate that KLF4 is increased *in vivo* during ECM we collected spleens from control IgG and anti-CD14 antibody treated mice on day 4 post-infection and isolated mRNA to quantify KLF4 expression by qRT-PCR (spleens are an important reservoir for monocytes that undergo trafficking to sites of inflammation [31]. Infected control IgG treated mice had a large increase in KLF4 mRNA expression compared to uninfected mice (Figure 4B). However, the increase in KLF4 expression is much less in infected monocyte depleted mice demonstrating that the increase in KLF4 during ECM is in part monocyte specific (Figure 4B; no change in uninfected IgG and CD14 antibody treated mice was seen, Supplementary Figure S4). This was confirmed on the protein level by pooling spleens from these mice then immunoprecipitating and immunoblotting for KLF4 (Figure 4C and Supplementary Figure S3).

### PF4 mediates an increase in KLF4 in vivo during ECM

**Figure 5:**
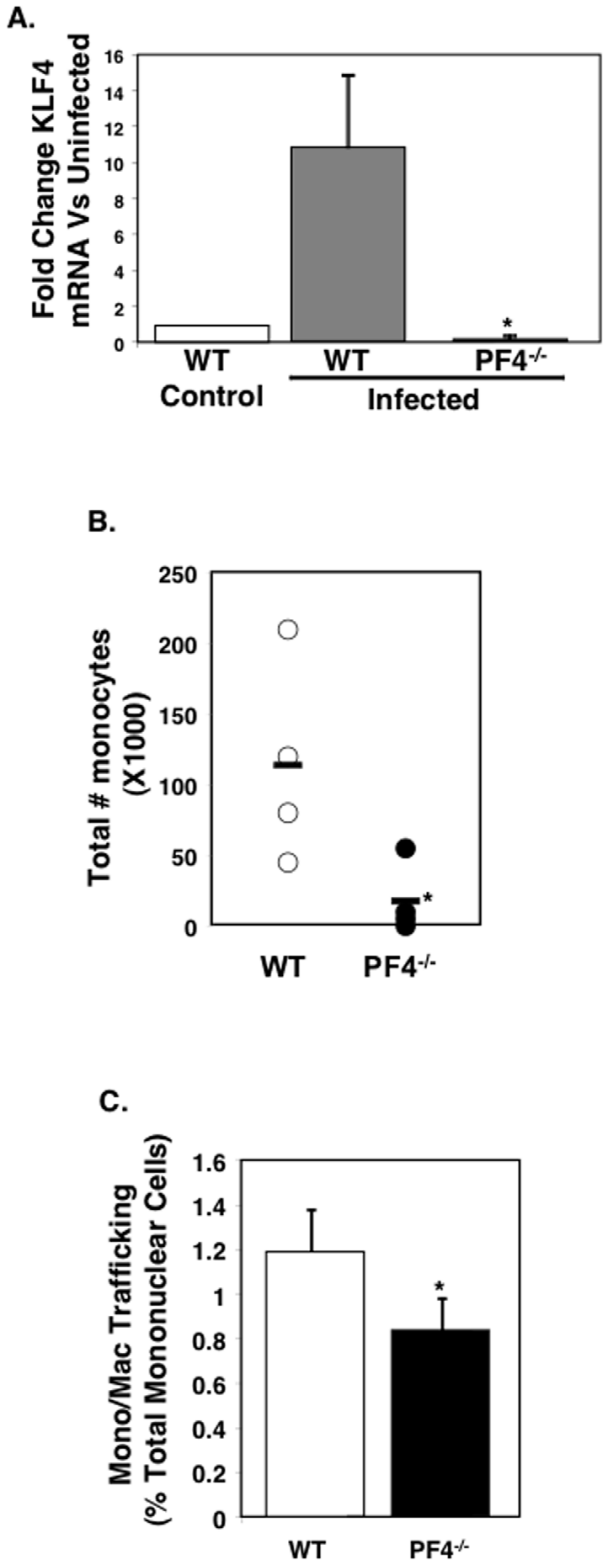
PF4 Mediates KLF4 Expression in ECM. A. qRT-PCR for KLF4 was performed on spleens from infected WT and PF4^−/−^ mice and expressed as fold change vs control uninfected mice (n = 5, S.D. *P<0.01 vs IgG). B. PF4 drives monocyte trafficking to the brain in ECM. Infected WT and PF4^−/−^ mouse brains were isolated on day 5 post infection and mononuclear cells isolated and total monocyte numbers determined (n = 5, ± S.D. *P<0.05). C. PF4 drives monocyte trafficking to the brain in ECM. Infected WT and PF4^−/−^ mice were given 1×10^6^ GFP positive monocytes on day 4, and 12 hrs later brains harvested and mononuclear cells isolated. GFP positive cells were quantified by FACS (n = 5, ± S.D. *P<0.03).

Our prior work has demonstrated that PF4^−/−^ mice have improved ECM survival and reduced plasma cytokines. This *in vitro* data demonstrated that PF4 increased monocyte KLF4 expression. To determine whether PF4 has a role in driving KLF4 expression during ECM we isolated spleens from infected WT and PF4^−/−^ mice on day 4 post-infection and quantified KLF4 expression by qRT-PCR. Similar to our results in Figure 4, WT mice had a greater than 4 times increase in KLF4 mRNA expression as compared to control uninfected mice (Figure 5A). However, PF4^−/−^ mice had very little change in KLF4 expression (Figure 5A), demonstrating a PF4-KLF4 signaling axis is present and functional in ECM.

Our prior work demonstrated that *P. berghei* infected PF4^−/−^ mice have reduced T-cell trafficking to the brain as compared to WT infected mice [10]. We have now found similar results with monocyte trafficking. WT and PF4^−/−^ mice were infected and on day 5 post infection brain mononuclear cells were isolated as we have described [10] and the total number of monocytes quantified using a mouse automated hematologic analyzer. Infected WT mice had a much greater number of brain monocytes compared to PF4^−/−^ mice (Figure 5B). To further demonstrate this is a direct PF4 effect on cell trafficking, mice were given GFP positive monocytes on day 4 post infection. Twelve hours later brains were collected from the mice, mononuclear cells isolated, and GFP positive cells quantified as a percent of total mononuclear cells by flow cytometry. WT mice had significantly greater monocyte trafficking to the brain as compared to PF4^−/−^ mice (Figure 5C) demonstrating an important role for PF4 in driving monocyte recruitment in ECM.

Taken together these data demonstrate that PF4 increases monocyte activation and KLF4 expression in vitro and in vivo in a cerebral malaria disease model.

## Discussion

Platelets are dynamic cells with important roles in hemostasis and inflammation. A more complete appreciation for platelet immune functions continues to become unraveled [3]. Work from our lab has demonstrated an important role for platelets, and platelet derived PF4, in T-cell recruitment and the pathogenesis of ECM [10]. This work now demonstrates that PF4 also helps drive an early innate immune response to *Plasmodium* infection by activating monocytes in a KLF4 dependent manner.

An important role for platelets in the pathogenesis of cerebral malaria is an area of increasing interest, and perhaps confusion. Platelets have been described to have diverse roles in vascular biology, likely a result of the diversity of inflammatory mediators platelets secrete or express upon activation. For example, platelets have been described to support angiogenesis [32], yet PF4 is a major platelet chemokine that has been described as an inhibitor of angiogenesis [11], [33], [34]. Our work, and that of many other investigators, has demonstrated that platelets have a deleterious role in ECM [5], [7], [8], [10], [27], [35]. However, intriguing recent work has indicated that the role of platelets may be dependent on the manifestation of *Plasmodium* infection by demonstrating that platelets can contribute to the killing of intraerythrocytic parasites in uncomplicated malaria [36]. These studies all point out the important complexity of platelet mediated signaling pathways and the need for further study to better understand the role of platelets in malaria pathogenesis.

PF4 makes up approximately 25% of the alpha granule content and reaches plasma concentrations of greater than 1 µg/mL in ECM [10], [11], [34]. PF4 has been most studied in the context of heparin induced thrombocytopenia during which PF4-heparin complexes are immunogenic and lead to antibody mediated platelet destruction [37]–[39]. Immune roles for PF4 are less well described, but likely more important given the global prevalence of vascular inflammatory diseases ranging from atherosclerosis to cerebral malaria. Elimination of PF4 reduces atherosclerotic lesion size in ApoE^−/−^ mice and disruption of PF4 and RANTES complexes can also reduce atherosclerosis in this mouse model [40], [41]. PF4 may also have a seemingly contradictory role by improving survival after LPS challenge [42]. Our work has shown that in the context of ECM PF4 has a deleterious role in driving immune stimulation, including monocyte activation.

The Kruppel like family of transcription factors have major disease relevance, particularly in cardiovascular pathologies [43]–[46]. The KLF family helps direct cell proliferation, differentiation, and survival in numerous tissue types ranging from gastrointestinal to immune cells [47]–[49]. The importance of KLF function is underscored by relatively small changes in KLF expression having large functional changes. For example hemizygous KLF2 mice have altered atherosclerotic risk and haploinsufficiency of KLF4 results in increased intestinal tumor formation [50], [51]. KLF4 can be a transcriptional activator or repressor depending on the promoter context and other transcription factor interactions [48], [52]. KLF4 is a critical transcription factor in controlling monocyte differentiation and is also essential for inflammatory monocyte development [25]. Over expression of KLF4 in pre-monoctye cell lines drives cell maturation to monocytes, and forced expression of KLF4 in myeloid progenitors or hematopoietic stem cells leads to monocyte differentiation [23], [24]. Our work demonstrates that PF4 leads to an upregulation of monocyte KLF4 expression and cytokine production in vitro and in vivo. Elaboration of cytokines is a major event in the development of cerebral malaria. Targeting PF4 induced KLF4 signaling networks may therefore be a target in interrupting the inflammatory pathways that lead to ECM.

Because KLF4 is vital in the development and differentiation of stem cells, KLF4^−/−^ mice are embryonic lethal. Reconstitution of WT mice with fetal derived KLF4^−/−^ heme progenitor cells is also problematic, as it results in mice with greatly suppressed monocyte numbers [23]. Unfortunately this means that direct evidence for the importance of PF4-KLF4 monocyte signaling in ECM is lacking from our work. However, our data clearly demonstrate that PF4 leads to monocyte activation that is at least in part a KLF4 dependent process. Infected PF4^−/−^ mice had approximately the same amount of KLF4 mRNA expression as uninfected mice, indicating that KLF4 is increased in ECM in a PF4 dependent manner. Because PF4^−/−^ mice are protected from ECM and have less monocyte trafficking this provides a strong link to the importance of PF4 driven KLF4 expression in ECM.

Like all disease model systems, the mouse model of cerebral malaria is imperfect. However, it does recapitulate many of the important immune aspects of cerebral malaria and is the best model system available to study the early steps in cerebral malaria pathogenesis [2], [53]. Although the parasite burden does not reach those levels found in humans, much of the immune cytokine dysfunction and brain histopathology are recapitulated [53]. In particular, a potential vital role for platelets in driving cerebral malaria and the presence of platelet aggregates in brain lesions has been demonstrated in both human cerebral malaria and the mouse model [6], [7].

Platelets are major mediators of the immune response, particularly in cerebral malaria. Despite much investigation cerebral malaria continues to be a persistent problem leading to great morbidity and mortality in the developing world. A better understanding of platelets and platelet derived mediators in ECM may lead to the development of new therapeutic strategies to treat this persistent clinical problem in much of the world.

## Materials and Methods

### Ethics Statement

All animal studies were performed at an AAALAC accredited institute using methods and guidelines approved by University of Rochester Animal Care and Use Committee that comply with United States national guidelines for the use of animals in research.

### Reagents

ELISA kits, KLF4 chromatin immunoprecipitation kit and recombinant mouse PF4 were purchased from R & D systems. Human PF4 was purchased from Haematologic Technologies. KLF4 and actin antibodies were purchased from Abcam. CD14 antibody was purchased from eBioscience. SiRNA and actin antibody were purchased from Santa Cruz Biotechnologies. CX3CR-GFP mice were purchased from Jackson Labs. Heparin, thrombin and hirudin were purchased from Sigma.

### Mouse infections

PF4^−/−^ mice are on a C57Bl6 background as we have used and reported in a past publication [10]. Mice were infected with *P. berghei* ANKA by injection of approximately 0.5×10^6^ parasites intraperitoneal (IP).

### In Vitro Monocyte Culture

For in vitro monocyte studies, C57Bl6/J mouse femurs were flushed to isolate bone marrow, single cell suspensions formed and red blood cells lysed with ACK lysis buffer. Monocytes were then isolated by negative selection (StemCell Technology) and incubated in DMEM with 5% FBS. THP-1cells were also maintained in the same media. GFP positive mouse monocytes were isolated using the same methods.

### PCR and Western blots

Cell or tissue samples for qRT-PCR were collected and stored in RNA Later (Ambion) solution at −80°C until RNA isolation using Trizol. 100 ng of RNA was used for Reverse Transcriptase (RT) reaction to make cDNA using Applied Biosystems High Capacity cDNA RT Kits and cDNA used for Real Time amplification analysis using a BioRad MyiQ Single Color Real Time PCR Detection system and Taqman Gene Expression mastermix (Applied Biosystem). The probes used were ordered from Applied Biosystems (Mm00607939_s1 mouse Actb, Mm00516104_m1 mouse KLF4, Hs99999903_m1 Human Actb, Hs00350836_m1 Human KLF4).

Western blot quantification was performed using a Bio-Rad Gel Doc Imaging Station and software.

KLF4 ChIP was performed using a KLF4 specific kit and amplification of bradykinin 2 receptor was with primers provided by the kit. Primers for amplification of IL-6 were; forward AGTGGTGAAGAGACTCAGTG and reverse GGCAGAATGAGCCTCAGA [54].

### Platelet Releasate Preparation

Washed WT and PF4^−/−^ mouse platelets were isolated and resuspended in Tyrode's buffer as we have described [55] and activated with 0.5 U/mL of thrombin (Sigma) for 10 mins before neutralization with hirudin (Sigma) and centrifugation. Isolated supernatant contained platelet releasate. Releasate from 2×10^6^ WT or PF4^−/−^ platelets was then added to 1×10^5^ monocytes for 24 hrs.

### Brain Mononuclear Cell Isolation

Brain mononuclear cells were isolated by removing brains and a single cell suspension obtained by grinding the tissue and mincing it with a razor blade in Dulbecco modified Eagle medium with 10% fetal bovine serum while on ice. Cell suspensions were placed in 15-ml conical tubes and Percoll (Sigma) added to a final concentration of 30%. One mL of Percoll was underlaid and cells spun at 1,300×*g* for 30 min at 4°C. Cells at the interface were isolated, washed twice, resuspended in Tyrode's buffer, and flow cytometry performed. Brain monocyte counting was performed using an Abaxis Veterinary Diagnostics VetScan HM5 Hematology System automated analyzer.

## Supporting Information

Figure S1 PF4 increases monocyte KLF4 expression. Blocking KLF4 by siRNA in THP-1 cells blocks IL-6 in response to PF4. Western Blot control.
(1.56 MB TIF)

Figure S2 CBC of control and CD14 antibody treated mice. Mice were treated with control IgG or anti-CD14 antibody IP and 12 hours later blood was collected for CBC (n = 4, *P<0.02).
(1.56 MB TIF)

Figure S3 KLF4 is increased in *P. berghei* infected mice. Mice were infected with *P. berghei* and on day 5 p.i. spleens were immunoblotted for KLF4 from infected mice and control uninfected mice. KLF4 is increased in the spleens of infected mice (top). Tubulin was used as loading control (bottom).
(1.56 MB TIF)

Figure S4 KLF4 mRNA is unchanged in uninfected anti-CD14 antibody treated mice compared to control IgG treated mice.
(1.56 MB TIF)
