# Supplementary figures and images for: Platelet Factor 4 Regulation of Monocyte KLF4 in Experimental Cerebral Malaria
Source: PLoS One. 2010 May 3;5(5):e10413. doi: 10.1371/journal.pone.0010413 (PMC2862712; doi:10.1371/journal.pone.0010413)

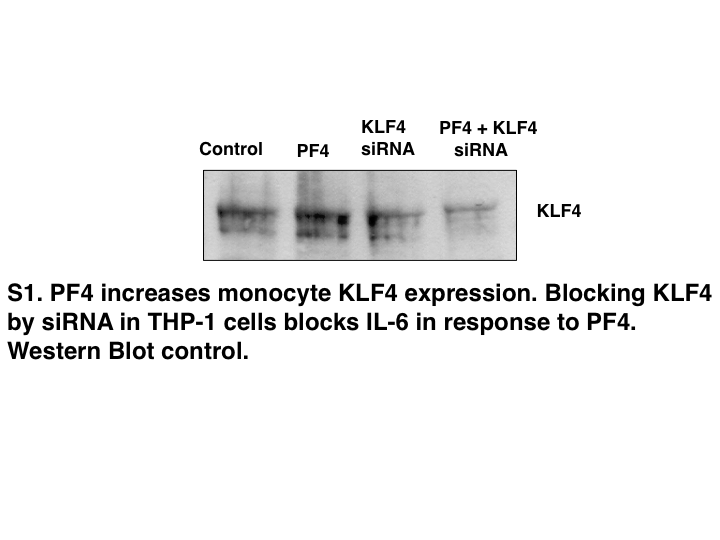

Supplement: Figure S1 — PF4 increases monocyte KLF4 expression. Blocking KLF4 by siRNA in THP-1 cells blocks IL-6 in response to PF4. Western Blot control. (1.56 MB TIF) [file pone.0010413.s001.tif]

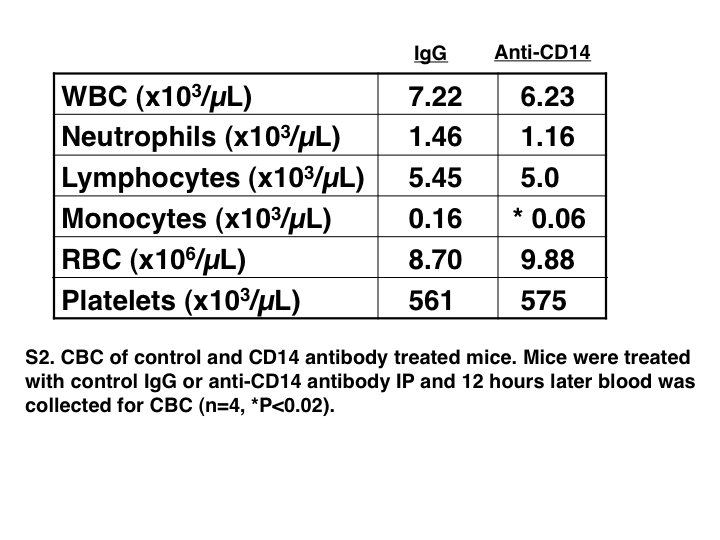

Supplement: Figure S2 — CBC of control and CD14 antibody treated mice. Mice were treated with control IgG or anti-CD14 antibody IP and 12 hours later blood was collected for CBC (n = 4, *P<0.02). (1.56 MB TIF) [file pone.0010413.s002.tif]

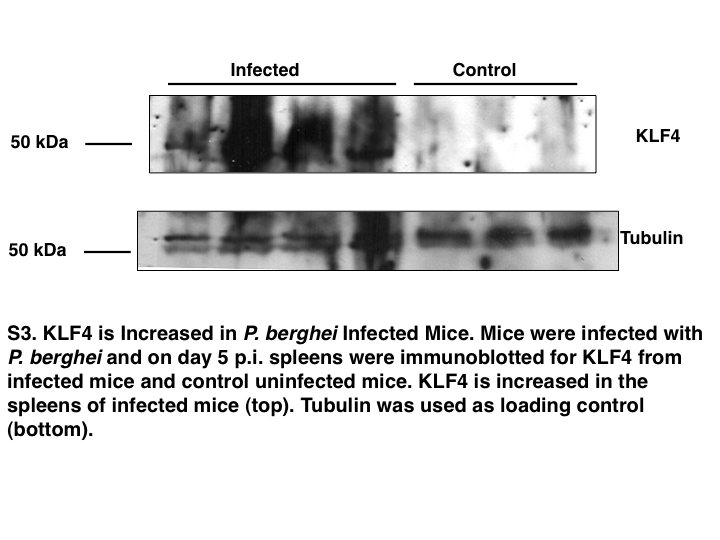

Supplement: Figure S3 — KLF4 is increased in P. berghei infected mice. Mice were infected with P. berghei and on day 5 p.i. spleens were immunoblotted for KLF4 from infected mice and control uninfected mice. KLF4 is increased in the spleens of infected mice (top). Tubulin was used as loading control (bottom). (1.56 MB TIF) [file pone.0010413.s003.tif]

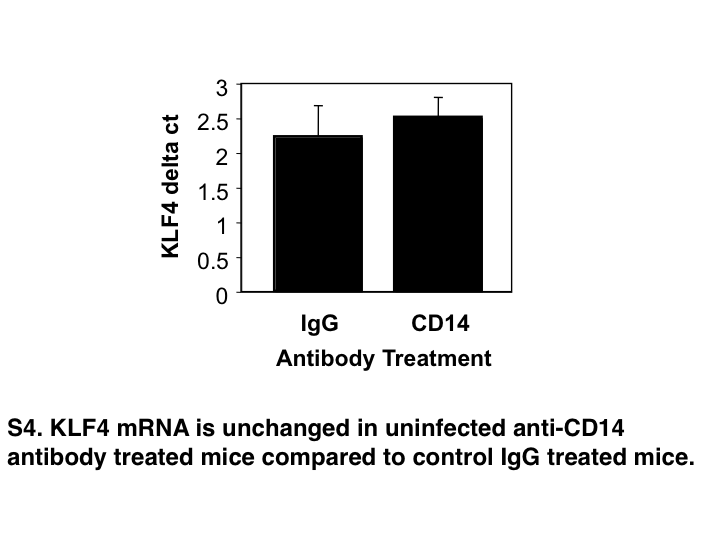

Supplement: Figure S4 — KLF4 mRNA is unchanged in uninfected anti-CD14 antibody treated mice compared to control IgG treated mice. (1.56 MB TIF) [file pone.0010413.s004.tif]
